# Mapping Microproteins and ncRNA-Encoded Polypeptides in Different Mouse Tissues

**DOI:** 10.3389/fcell.2021.687748

**Published:** 2021-07-26

**Authors:** Ni Pan, Zhiwei Wang, Bing Wang, Jian Wan, Cuihong Wan

**Affiliations:** Hubei Key Laboratory of Genetic Regulation and Integrative Biology, School of Life Sciences, Central China Normal University, Wuhan, China

**Keywords:** small open reading frame, non-coding RNAs, *de novo* sequencing, top-down, mouse tissue

## Abstract

Small open reading frame encoded peptides (SEPs), also called microproteins, play a vital role in biological processes. Plenty of their open reading frames are located within the non-coding RNA (ncRNA) range. Recent research has demonstrated that ncRNA-encoded polypeptides have essential functions and exist ubiquitously in various tissues. To better understand the role of microproteins, especially ncRNA-encoded proteins, expressed in different tissues, we profiled the proteomic characterization of five mouse tissues by mass spectrometry, including bottom-up, top-down, and *de novo* sequencing strategies. Bottom-up and top-down with database-dependent searches identified 811 microproteins in the OpenProt database. *De novo* sequencing identified 290 microproteins, including 12 ncRNA-encoded microproteins that were not found in current databases. In this study, we discovered 1,074 microproteins in total, including 270 ncRNA-encoded microproteins. From the annotation of these microproteins, we found that the brain contains the largest number of neuropeptides, while the spleen contains the most immunoassociated microproteins. This suggests that microproteins in different tissues have tissue-specific functions. These unannotated ncRNA-coded microproteins have predicted domains, such as the macrophage migration inhibitory factor domain and the Prefoldin domain. These results expand the mouse proteome and provide insight into the molecular biology of mouse tissues.

## Introduction

Microproteins are short peptides translated by mRNAs or non-coding RNAs (ncRNAs) with small open reading frames (sORFs, shorter than 100–150 codons; Ingolia, 2014; Ma et al., 2016). sORFs are widely distributed throughout the genomes of all species, such as human, mouse, and fruitfly (Frith et al., 2006). With the development of ribosome profiling, mass spectrometry (MS), and bioinformatics, increasing number of sORF-encoded microproteins have been discovered. It has been reported that ncRNAs tend to encode low molecular weight proteins (Lu et al., 2020). Furthermore, they could play essential roles in development, muscle function, and metabolism (Kondo et al., 2007; Magny et al., 2013; Lee et al., 2015). Together with microproteins encoded by mRNA, the polypeptides encoded by ncRNAs are particularly important (Jackson et al., 2018). Several polypeptides encoded by ncRNAs had fundamental functions in muscle regeneration and tumor development (Nelson et al., 2016; Huang et al., 2017). For example, the 34 amino acid (AA) peptide DWORF promotes muscle formation (Nelson et al., 2016); a 59 AA peptide SMIM30 induces cell proliferation and migration of liver cancer (Pang et al., 2020); and the 60 AA peptide SPRS inhibits angiogenesis in breast cancer (Wang et al., 2020).

ncRNAs have been found to exhibit tissue-specific expression (Landgraf et al., 2007; Liang et al., 2007; Cabili et al., 2011), suggesting that they can carry out tissue-specific functions. For example, a liver-enriched long non-coding RNA, lncLSTR, regulates systemic lipid metabolism in mice (Li et al., 2015). In the past year, researchers have provided some tissue-specific uncharacterized ncRNAs in various tissues that may be involved in health and disease (Isakova et al., 2020). Meanwhile, more and more ncRNAs are proved to be capable of coding (Ruiz-Orera et al., 2014). For example, peptide MLN encoded by a putative long non-coding RNA regulates muscle performance (Anderson et al., 2015). The study of ncRNA-encoded polypeptides in various tissues may significantly help us understand their functions. To study functional microproteins in different tissues, these microproteins must first be identified. However, large-scale microprotein identification has only been applied to a few tissues, such as the brain (Li et al., 2017; Budamgunta et al., 2018) and heart (van Heesch et al., 2019). This inspired us to systematically study the distribution of microproteins and ncRNA-encoded polypeptides among tissues.

Mass spectrometry was used to identify microproteins because of the direct detection of translated products. Bottom-up proteomic strategy involving specific sample preparation was the primary method used to search for microproteins. For example, Slavoff et al. (2013) detect sORF-encoded polypeptides (SEPs) in the human K526 cell line and identified 90 microproteins, 86 of which are previously uncharacterized. Using a similar method, 117 microproteins were identified in *Saccharomyces cerevisiae* (He et al., 2018). Our group identified 271 microproteins in the Hep3B cell line (Wang et al., 2021a). Researches have also used a digestion-free top-down strategy and database-independent *de novo* sequencing to identify microproteins (Hughes et al., 2010; Li et al., 2017; Wang et al., 2021b). Here, we used a combination of bottom-up, top-down, and *de novo* sequencing methods to identify microproteins in five mouse tissues. As a result, we found 1,074 microproteins, 270 of which were ncRNA-encoded, and 556 of which were tissue-specific. Nearly half of these microproteins have no MS or translation evidence according to the OpenProt database.

## Materials and Methods

### Tissue Preparation

BALB/c mice were obtained from the Hubei Center for Disease Control. The 11-week-old mice were sacrificed by cervical dislocation. Tissues were removed from the mice, washed with cold phosphate-buffered saline (PBS) to remove residual blood, and stored in a freezer at −80°C until further use. All animal experiments were conducted following the guidelines provided by the Institutional Animal Care and Use Committee of Central China Normal University.

### Protein Extraction

From each sample, 100 mg of tissue were used. Microproteins were extracted using HCl and RIPA buffers separately, as described in the following steps:

Boiling water (200 μL) was added to the samples and left to boil for 10 min. Then, we took the aqueous components into a new centrifuge tube and added 500 μL of HCl buffer [50 mM HCl, 0.5% dithiothreitol (DTT)] to each sample. The samples were homogenized in a Dounce homogenizer (Kimble, Manzanillo, Mexicos) and centrifuged at 12,000 × *g* and 4°C for 30 min. The supernatant was collected and mixed with water-containing parts. Then, 125 μL of chloroform and 500 μL of ddH_2_O were added to each sample, mixed vigorously, centrifuged at 12,000 × *g* for 10 min at room temperature, and the supernatant was transferred to a new centrifuge tube. The supernatant was then dried under vacuum at a low temperature in a SpeedVac (Labconco, KS, United States).

On the other way, 1 mL RIPA buffer [150 mM NaCl, 50 mM Tris–HCl, 5 mM sodium fluoride, 1 mM sodium orthovanadate, 0.1% SDS, 1% NP40, 1 EDTA-free protease inhibitor tablet (Roche, Mannheim, Germany) per 10 mL of lysis buffer] was added to each tissue sample, homogenized in a Dounce homogenizer on ice, and centrifuged at 12,000 × *g* for 30 min at 4°C. The supernatant was collected, 250 μL chloroform and 1 mL ddH_2_O were added. The mixture was mixed vigorously and centrifuged at 12,000 × *g* for 10 min at room temperature. The supernatant was transferred to a new centrifuge tube. The BCA assay was used to quantify the protein amount of each sample.

### SDS-PAGE

The samples extracted with HCl buffer were resuspended with 50 mM NH_4_HCO_3_, separated by 16% tricine sodium dodecyl sulfate-polyacrylamide gel electrophoresis (SDS-PAGE) and stained with Coomassie blue.

The samples extracted with RIPA buffer were resuspended in 50 mM NH_4_HCO_3_ and separated by 12% Glycine SDS-PAGE and stained with Coomassie blue. Each lane was sectioned below the 25 kDa marker into six sections followed by in-gel trypsin digestion.

### Trypsin Digestion

The dried protein mixtures (100 μg each) were resuspended in 50 mM NH_4_HCO_3_, then incubated with DTT at a final concentration of 10 mM at 37°C for 1 h, and alkylated with 15 mM iodoacetamide (IAM) in the dark at room temperature for 30 min. Each sample was then incubated with 2 μg trypsin at 37°C for 16 h. The enzymatic digestion was stopped with formic acid at final concentration of 5%.

Each gel slice was washed with 500 mL of 50% acetonitrile (ACN)/50 mM NH_4_HCO_3_ (pH 8.0) for 10 min. This process was repeated three times. Gel slices were dried under vacuum at low temperature in a SpeedVac for 5 min, incubated in 10 mM DTT/50 mM NH_4_HCO_3_ at 56°C for 1 h, and then incubated in 50 mM IAM/50 NH_4_HCO_3_ at 37°C for 45 min in the dark. Then, the gel slices were washed with ACN and vacuum-dried for 5 min. Digestion was then performed at 37°C for 16 h with 0.02 μg/μL trypsin in 50 mM NH_4_HCO_3_. Peptides were extracted with 60% ACN/5% formic acid for 10 min, and the supernatant was collected into a new tube and vacuum-dried. All digested peptide mixtures were desalted on a C18 StageTips (3M Empore^TM^, St.Paul, MN, United States).

### Protein Fractionation

Non-enzymatic samples extracted with HCl buffer were resuspended in 200 μL NH_4_FA. Proteins were separated by 5–70% B (mobile phase A: 25 mM NH_4_FA water; mobile phase B: 25 mM NH_4_FA acetonitrile), with a homemade C18 column (3M Empore^TM^). Eight fractions were collected per sample and directly analyzed using MS.

### Synthesized Peptides

Peptides were synthesized using standard Fmoc chemistry in the Guo Tai bio-company (www.bankpeptide.com, Hefei, China). The sequences of peptides are LPLPLGR, LLEPSLR, FNPDVSWDR, NVLEEEGR, FVSEAELDER, GLFLLDDK, LAVAAQNCYK, and NDVFVLEEWGR. The peptides were resuspended in 5% ACN. The peptide loading amount was 1 pmol. The mass spectrum parameters and database search were consistent with those of the digestion sample.

### LC/MS/MS Analysis

The peptides were resuspended in 0.1% formic acid water and analyzed using the Q Exactiv^TM^Plus Orbitrap high-resolution mass spectrometer and the EASY nLC^TM^1200 system (Thermo Fisher Scientific, Bremen, Gemany). The samples were separated on a homemade C18 column (15 cm, 75 μm, 3 μm, and 100 Å) at a flow rate of 0.5 μL/min. The peptides were separated by 120 min 5–80% B (mobile phase A: 0.1% formic acid water; mobile phase B: 0.1% formic acid ACN). MS analysis use data-dependent acquisition mode with parameters settings: full MS [automatic gain control (AGC), 3 × 10^6^; resolution, 7 × 10^4^; m/z range, 350–20,000; and maximum ion time, 20 ms]; MS/MS (AGC, 5 × 10^4^; maximum ion time, 50 ms; minimum signal threshold, 4 × 10^3^; dynamic exclusion time setting, 40 s; and unassigned and singly charged ions were excluded).

The MS parameters for non-enzymatic samples were the same as those mentioned above, except for the following settings: full MS (AGC, 5 × 10^6^; maximum ion time, 200 ms); MS/MS (AGC, 1 × 10^6^; maximum ion time, 200 ms; dynamic exclusion time setting, 45 s; unassigned, singly, and double charge ions were excluded).

All LC/MS/MS raw data related to this work will be uploaded to iProX^1^ and available for download with access ID IPX0002949000.

### Data Analysis

Raw files obtained by the bottom-up method were analyzed using Proteome Discoverer v2.1 (Thermo Fisher Scientific, Rockford, United States) with the following parameters: enzyme, trypsin; missed cleavage, 2; precursor mass tolerance, 10 ppm; fragment mass tolerance, 0.02 Da; methionine oxidation and N-terminal acetylation as dynamic modification, carbamidomethylation as a static modification. The false discovery rate (FDR) was set to 1%. The protein fasta file used mouse protein data from OpenProt^2^, which contains RefProts, Isoforms, and AltProts.

Data analysis for *de novo* sequencing was performed as follows. Raw files were converted to mgf files using MSConvertGUI and analyzed by pNovo v3.1^3^ with the following parameters: enzyme, trypsin; precursor mass tolerance, 10 ppm; fragment mass tolerance, 0.02 Da; methionine oxidation and N-terminal acetyl as dynamic modifications; carbamidomethylation as a static modification; open search, true; keep results, top-1. For one spectrum, if *de novo* sequencing yielded the same results as the data-dependent search, we considered it a positive result. However, if *de novo* sequencing differed from the data-dependent search, we considered the *de novo* sequencing result as a false positive. If the spectrum was not assigned to any peptide in the data-dependent search but assigned to a peptide in *de novo* sequencing, we considered it a new spectral peptide segment. Based on the precision-recall curves (Supplementary Figure 1), the optimized cutoff score was 72. Peptides with scores > 72 are remained as confident *de novo* sequencing results. The software ACTG (Choi et al., 2017) was used to map peptide sequences onto genome sequences. The Proteogenomic mapping tool (Sanders et al., 2011) was used to lookup these peptide segments’ open reading frames on the genome.

Raw data of non-enzymatic samples were analyzed using pFind v3.1^4^ with the following parameters: enzyme, no enzyme; precursor tolerance, 10 ppm; fragment tolerance, 0.02 Da; FDR set to 1%. The protein database used mouse protein data from OpenProt.

The length of microprotein was defined as less than 50, 100, or 150 amino acid in different researches (Slavoff et al., 2013; Storz et al., 2014; Ma et al., 2016). In this work, we use 150 AA as a cutoff.

### Bioinformatic Analysis of Identified Microproteins

Gene ontology (GO) enrichment analysis of microproteins was processed using DAVID online bioinformatics tools^5^. Microprotein domain analysis was performed using the Pfam search tool^6^. Protein-protein interactions were obtained from the STRING database^7^.

## Results and Discussion

### Workflow of Microproteins Identification in Mouse Tissue

**FIGURE 1:**
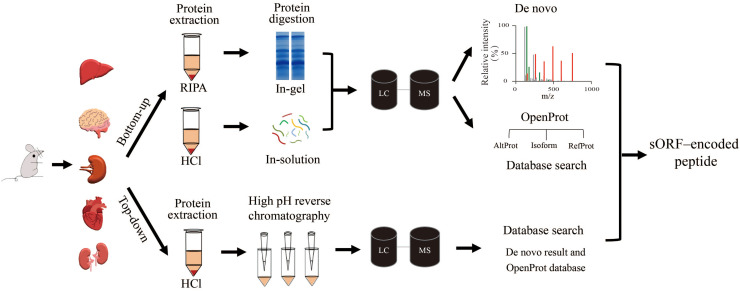
Workflows tested in the discovery of microproteins in different tissues. Sample preparation for the bottom-up method used two different lysis buffers: RIPA and HCl. The protein mixtures were then digested and subjected to LC/MS/MS. After that, the mass spectra were searched against the fasta file that was downloaded from the OpenProt database. Meanwhile, *de novo* sequencing directly determained the peptide sequence according to the spectrum without a fasta file. The top-down protein samples were fractionated with high pH reverse chromatography before LC/MS/MS analysis. The mass spectra were searched against a fasta file containing proteins from the OpenProt database and polypeptides from *de novo* results.

**FIGURE 2:**
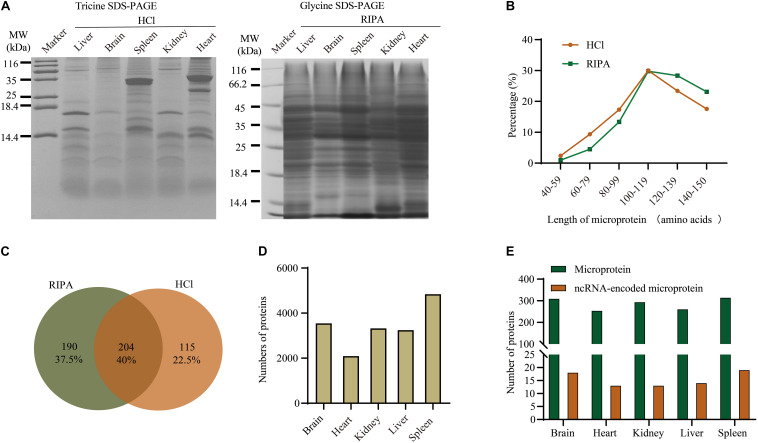
Overview of proteins identified in different tissues using the bottom-up method and database search. **(A)** Separation of total proteins from five tissues using glycine SDS-PAGE and tricine SDS-PAGE. **(B)** Comparison of microprotein lengths using different buffers. **(C)** Venn diagram of microproteins identified using RIPA and HCl buffers. **(D)** Numbers of detected proteins in different tissues. **(E)** Distribution of microproteins and ncRNA-encoded microproteins among five tissues.

We selected five mouse tissues (brain, heart, liver, spleen, and kidney) to investigate tissue-specific microproteins (<150 AA). The complexity of a complete proteome makes it challenging to detect all expressed microproteins because of their short lengths and low abundance (Khatun et al., 2013). To boost the identification of microproteins, we combined three MS methods, bottom-up, top-down, and *de novo* sequencing (Figure 1). Because *de novo* sequencing uses the dataset from the bottom-up method, the bottom-up method in this work refers to the method with trypsin digestion sample preparation and database-dependent searching. For the bottom-up strategy, RIPA lysis buffer and HCl lysis buffer (defined as RIPA and HCl, respectively) were used for protein extraction. Protein mixtures were separated by glycine or tricine SDS-PAGE to reduce the sample complexity (Figure 2A). Using this bottom-up and database dependent search approach, we identified 7,120 proteins in total from all samples (Supplementary Table 1).

The length distribution of microproteins (Figure 2B) showed that the RIPA extraction method favored proteins with a higher molecular weight than HCl extraction. This is because the HCl buffer was supposed to precipitate larger proteins in order to extract low molecular weight microproteins (Ma et al., 2016). Finally, 319 and 394 microproteins were identified from HCI and RIPA, respectively, and 204 microproteins were identified by the two methods (Figure 2C). These microproteins were unequally distributed among the five tissue types. Spleen sample with the most proteome identified also contained more microproteins and ncRNA-encoded microproteins, while heart tissue had less proteome and microproteins (Figures 2D,E).

### *De novo* Sequencing Provides Information Regarding New Microproteins and Related sORFs

**FIGURE 3:**
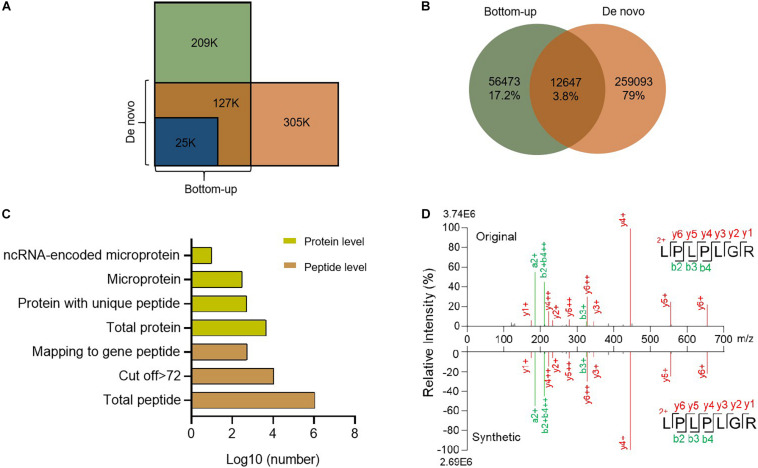
Summary of *de novo* sequencing results. **(A)** Comparison of spectrum counts between *de novo* sequencing and bottom-up database search. **(B)** Venn diagram of peptides between *de novo* sequencing and bottom-up. **(C)** Identification of peptides and proteins from *de novo* sequencing. **(D)** Comparison of the spectra for the *de novo* search and synthetic peptide.

*De novo* sequencing obtains peptide sequences directly from the LC/MS/MS spectra, which are not limited by sequences in any database (Hughes et al., 2010). There may still be plenty of unknown gene products, especially ncRNA-encoded proteins, not in the database. So we searched our LC/MS/MS raw files with the *de novo* sequencing technique. In total, we found about 400 k spectra from MS, among which 152 k were interpreted by database dependent search and 305 k by *de novo* sequencing (Figure 3A). These spectra correspond to 200 k new peptides (Figure 3B). To obtain high confidence peptides, we filtered the *de novo* results with a score > 72 (Supplementary Figure 1). After that, we got 11,097 peptides from 15,158 spectra, including 550 peptides that corresponded to open reading frames in the mouse genome. These peptides belong to 526 proteins (Supplementary Table 2), including 290 microproteins (Figure 3C and Supplementary Figure 2). Among these microproteins, 121 are novel gene coding products named Denovo001 to Denovo121 (Supplementary Table 2). A total of 104 *de novo* microproteins were tissue-specific.

To further confirm the quality of the *de novo* sequencing data, we randomly selected certain peptides for synthesis. We compared the spectra of the synthesized peptides with the *de novo* results and found them to be highly consistent (Figure 3D and Supplementary Figure 3), which suggested that our results were reliable. *De novo* sequencing identified new peptides or proteins that were not annotated in any current database (Yang et al., 2019). These novel peptides might have important functions.

### High Sequence Coverage of Microproteins Identified by Top-Down Approach

**FIGURE 4:**
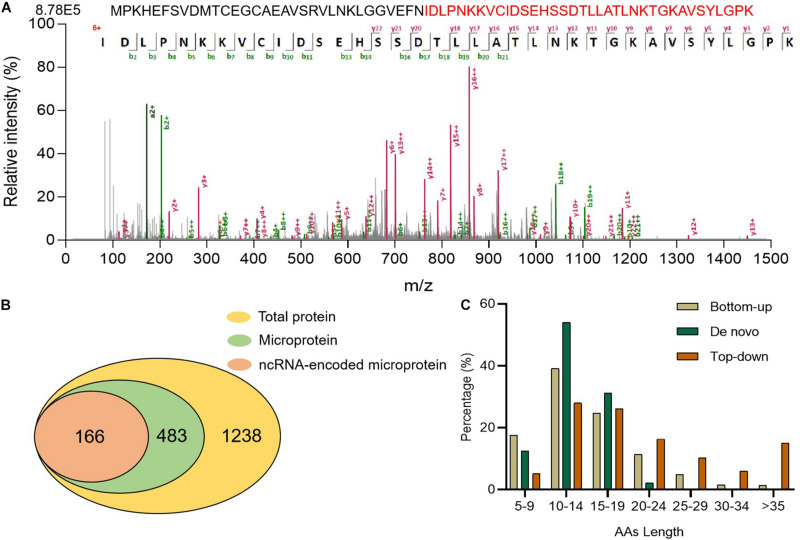
Proteins detected using the top-down strategy. **(A)** Full length O08997 and its MS/MS spectrum **(B)** Numbers of identified total proteins, microproteins, and ncRNA-encoded microproteins with unique peptides. **(C)** Comparison of microprotein AA lengths identified using three strategies.

Top-down is another helpful tool for identifying microproteins (Breuker et al., 2008; Ahlf et al., 2012) because it is superior to complete sequence analysis of intact protein (Cupp-Sutton and Wu, 2020). To improve the sequence coverage of microproteins, we adopted a top-down method. Without trypsin digestion, longer peptides could be identified, for example, a peptide of O08997 (Figure 4A). This microprotein contains 68 AA, and we identified its peptide with 36 AA, which provides 52.9% sequence coverage. This microprotein was also identified via the bottom-up strategy, but the coverage was only 11.8%. It was shown that the top-down strategy could greatly improve the coverage of peptide sequences. Using a top-down strategy, we detected a total of 1,238 proteins distributed among different tissues (Supplementary Table 3 and Supplementary Figure 4), including 483 microproteins and 166 encoded by ncRNA (Figure 4B). We also discovered a number of novel tissue-specific microproteins, such as IP_2438407 in the spleen and IP_1072519 in the heart, which have not been detected before. By comparing the top-down results with bottom-up and *de novo* sequencing results, we found that the length and the sequence coverage of the identified polypeptides were higher in the top-down approach (Figure 4C and Supplementary Figure 5). These results confirmed that the top-down strategy was more effective for longer peptide identification, thus increasing sequence coverage.

### Characteristics of Microproteins Identified in Five Mouse Tissues

Combining bottom-up, *de novo* sequencing, and top-down results, we identified 7,922 proteins in total, of which 1,074 were microproteins (Supplementary Table 4). These three methods collectively identified only 19 microproteins, which suggested excellent complementarity among them (Supplementary Figure 6).

**FIGURE 5:**
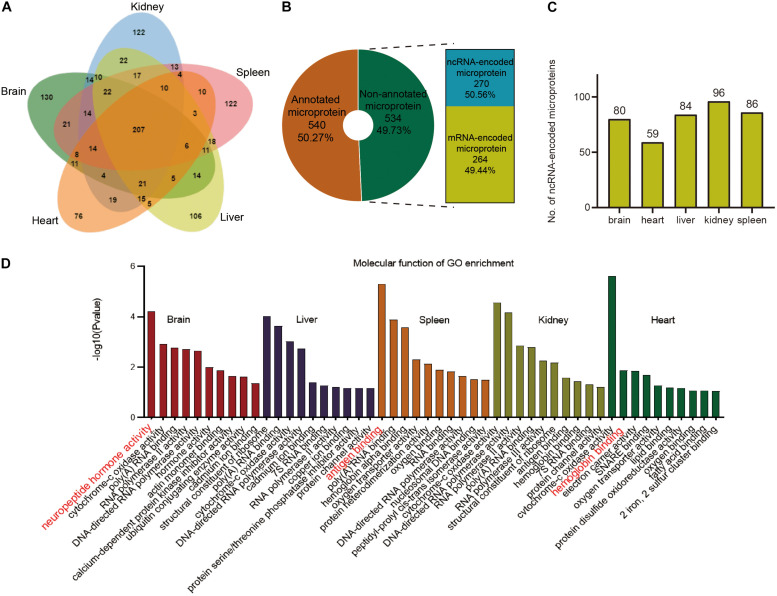
Overview of total microproteins and ncRNA-encoded microproteins. **(A)** Venn diagram of total microproteins identified in different tissues. **(B)** Distribution of annotated and non-annotated microproteins and the proportion of ncRNA-encoded non-annotated microproteins. **(C)** Distribution of ncRNA-encoded proteins across different tissues. **(D)** Gene ontology (GO) enrichment of the identified annotated microproteins.

A total of 207 microproteins were found in all five tissues, 518 in more than one tissue, and 556 in only one tissue (Figure 5A and Supplementary Figure 7). Brain tissue had the most tissue-specific microproteins, while the heart tissue had the least specific microproteins. 61% of the microproteins had MS evidence, and 54.2% had translation evidence according to the OpenProt database (Supplementary Figure 8). We found many novel microproteins in various tissues of mice, indicating that the data in our study can enrich the proteomic data of mice. Over half of these microproteins had predicted domains (Supplementary Figure 8), suggesting that they may have specific functions. However, half of them were non-annotated, of which 270 were encoded by ncRNAs (Figure 5B). Finally, 270 ncRNA-encoded microproteins were identified in the different tissues (Figure 5C and Supplementary Table 5). Kidney tissue had the highest number of ncRNA-encoded microproteins, and heart tissue contained minimal ncRNA-encoded microproteins. Although the focus was on microproteins, we also identified 116 ncRNA-encoded polypeptides larger than 150 AA (Supplementary Table 5). The length distribution of the ncRNA-encoded polypeptides showed that 79% of the proteins were greater than 50 AA in length (Supplementary Figure 9).

To explore the functions of the annotated microproteins in various tissues, we performed the GO enrichment analysis. The results indicated that microproteins in different tissues were mostly related to tissue-specific functions (Figure 5D). For example, brain tissue has many microproteins related to nerves and hormones, which is consistent with previous research findings (Marcus et al., 2004; Davis et al., 2018). Splenic tissue was rich in immunity-related microproteins, which is consistent with previous research (Hu et al., 2018; Ma et al., 2019). Representative microproteins with tissue-specific functions and domains are presented in Table 1. From our data, we found a microprotein named Neuromedin-B specifically expressed in the brain tissue. This protein in UniProt was inferred by homology and has a Bombesin domain, which is very important in mouse brain development (Secher et al., 2016). Interestingly, a negative regulator of P-body association was identified in brain, spleen and kidney tissues. This protein has high sequence similarity with Nobody (>80%), which was originally thought to be encoded by ncRNA, and plays an essential role in regulating mRNA processing (D’Lima et al., 2017).

**TABLE 1:** Part of the microproteins.

Tissues	Protein name	AA	Gene type	Exist level	Domain	Predict functions
Brain	Neuromedin-B	131	mRNA	Inferred from homology	Bombesin	Neuropeptide signaling pathway
	IP_970184	114	ncRNA	Three-frame translation	MIF	Nervous development
Heart	Ventricular/cardiac muscle isoform	93	mRNA	Experimental evidence	EF hand	Calcium ion binding
Liver	Apolipoprotein C-IV	124	mRNA	Experimental evidence	APOC4	Positive regulation of sequestering of triglyceride
Spleen	IP_991787	87	ncRNA	Three-frame translation	Prefoldin subunit	Chaperone
Common	IP_989670	64	ncRNA	Three-frame translation	Ubiquitin domain	Degradation of protein
	Negative regulator of P-body association	68	mRNA	Protein predicted	none	Negative regulation of cytoplasmic mRNA

### Tissue-Specific Microproteins Coded by ncRNAs

**FIGURE 6:**
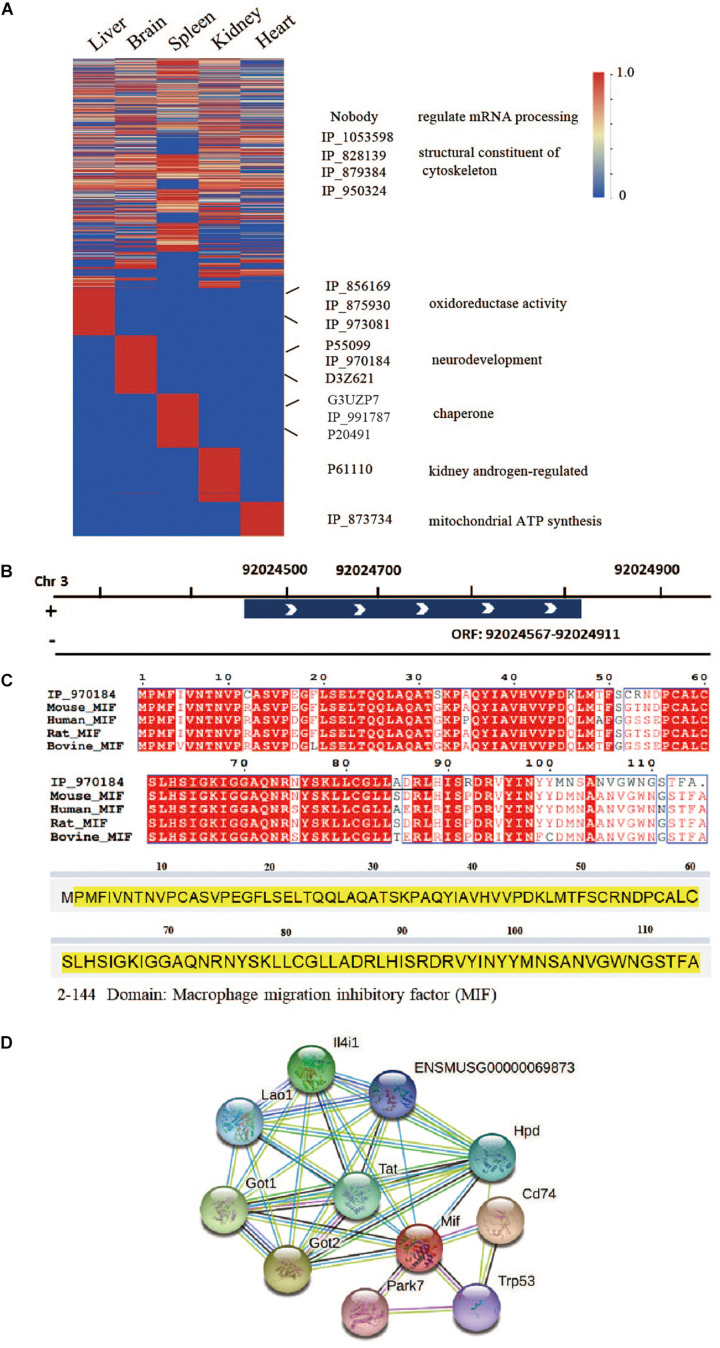
Tissue-specific microproteins. **(A)** Microprotein expression across different tissues (normalized spectral counts). **(B)** Location of IP_970184 on chromosome 3. **(C)** Sequence similarity between IP_970184 and macrophage migration inhibitory factor (MIF) across different species. The identified peptides of IP_970184 are labeled in black, and the domain sequence is highlighted in yellow. **(D)** Protein interactions of mouse macrophage migration inhibitory factor (MIF) from STRING database.

To determine the distribution of microproteins in various tissues, we performed label-free quantification with spectra counts (Figure 6A). So far, studies regarding SEPs have mainly focused on the identification of these peptides, and very few reports provide quantitative data concerning changes in SEP expression under different biological conditions (Cao et al., 2020; Fabre et al., 2021). Microprotein identification often has a low number of identification and low reproducibility (Cardon et al., 2020), which restricts the progress of quantification. Therefore, in this work, we only presented rough quantification results using spectral counts. From the results, we found that several microproteins, such as IP_1053598, IP_828139, IP_879384, and IP_950324, exist in multiple tissues. These four microproteins are ncRNA-encoded and structural constituents of the cytoskeleton. Some ncRNA-encoded microproteins, which may have important functions, were only identified in one tissue. To identify potential functions, we analyzed the domains of microproteins with PFAM online software^8^. According to domain analysis, liver-specific microproteins, IP_856169, IP_875930, and IP_973081 have glyceraldehyde-3-phosphate dehydrogenase domains. IP_873734 is specifically expressed in the heart and has a mitochondrial ATP synthesis domain.

One interesting ncRNA-encoded microprotein IP_970184, is only found in brain tissue (Supplementary Figure 10). Its open reading frame is located on chromosome 3 (Figure 6B). This microprotein had a macrophage migration inhibitory factor (MIF) domain and quite high sequence similarity to protein MIF. Furthermore, the microprotein was also conservative across 4 species (Figure 6C). Several proteins would interacts with MIF (Figure 6D), such as aspartate aminotransferase, which is encoded by *Got1* gene. *Gotl* is an important regulator of glutamate levels, acting as a glutamate scavenger in brain neuroprotection (Daikhin and Yudkoff, 2000). Previous studies have confirmed that MIF is critically involved in anxiety, depression, and memory-related behaviors. In addition to exerting a pro-inflammatory function, MIF expression is related to adult hippocampal neurogenesis (Conboy et al., 2011). Therefore, we believe that this ncRNA-encoded microprotein IP_970184 might have a regulatory function in the nervous system.

Another ncRNA-encoded microprotein, IP_991787, was only found in spleen tissue (Supplementary Figure 11). There is no mass spectrum or translation evidence for this microprotein in OpenProt database. Sequence alignment suggested that it has high sequence homology (>90%) to prefoldin subunit 6 with a prefoldin domain and conserved across four species (Supplementary Figure 12). The prefoldin complex is chaperone protein with multiple functions (Liang et al., 2020). A recent study proved that one novel prefolding-like microprotein, ASDURF, is a subunit of the PAQosome, which is a chaperone complex related to the biogenesis of plenty protein complexes (Cloutier et al., 2020). These results demonstrated that microprotein IP_991787 might have similar functions.

There are other interesting ncRNA-encoded polypeptides in our data, such as 88 AA IP_988951, which is expressed in all five tissues and contains NAD binding domain. Therefore, it may participate in the tricarboxylic acid cycle. Our dataset may provide useful information for future functional studies.

## Conclusion

In total, we detected 1,074 microproteins in five mouse tissues. There were 556 tissue-specific microproteins in various tissues. Brain tissue had the highest number of microproteins related to nerves and hormones; spleen and kidney tissues contained more immune-related microproteins. At the same time, we have also found 386 ncRNA-encoded polypeptides, 270 of which are microproteins. Some ncRNA-encoded microproteins have functional domains or are conserved across species, indicating that these microproteins might have important functions. Our protein express dataset was only based on MS quantification. It will be better to validate the microprotein expression by western blot with specific antibodies. However, we have presented a large-scale survey of microproteins encoded by mRNA or ncRNA and mined these data to better understand the biochemical basis of tissue specificity. These results will hopefully stimulate future microprotein and ncRNA-encoded microprotein studies involving different tissues and organisms.

## Data Availability Statement

The original contributions presented in the study are publicly available. This data can be found here: Integrated proteome resources accession, project ID IPX0002949000, ProteomeXchange ID PXD025158 https://www.iprox.org/page/project.html?id=IPX0002949000.

## Ethics Statement

The animal study was reviewed and approved by Institutional Animal Care and Use Committee Central China Normal University.

## Author Contributions

NP performed the experiments with the association of BW and JW, and wrote the manuscript. ZW did the *de novo* data analysis with NP. CW supervised the project, designed experiments, and wrote the manuscript. All authors read and approved the final manuscript.

## Conflict of Interest

The authors declare that the research was conducted in the absence of any commercial or financial relationships that could be construed as a potential conflict of interest.

## Publisher’s Note

All claims expressed in this article are solely those of the authors and do not necessarily represent those of their affiliated organizations, or those of the publisher, the editors and the reviewers. Any product that may be evaluated in this article, or claim that may be made by its manufacturer, is not guaranteed or endorsed by the publisher.

## Footnotes

1 http://www.iprox.org

2 http://openprot.org/

3 http://pfind.ict.ac.cn/software/pNovo/index.html

4 http://pfind.ict.ac.cn/software/pFind/index.html

5 https://david.ncifcrf.gov/tools.jsp

6 http://pfam.xfam.org/search

7 https://string-db.org/

8 http://pfam.xfam.org
