# Supplementary material for: Mapping Microproteins and ncRNA-Encoded Polypeptides in Different Mouse Tissues
Source: Front Cell Dev Biol. 2021 Jul 26;9:687748. doi: 10.3389/fcell.2021.687748 (PMC8350139; doi:10.3389/fcell.2021.687748)
Supplement: Supplementary file 1 [file Data_Sheet_1.docx]

## Supplementary information


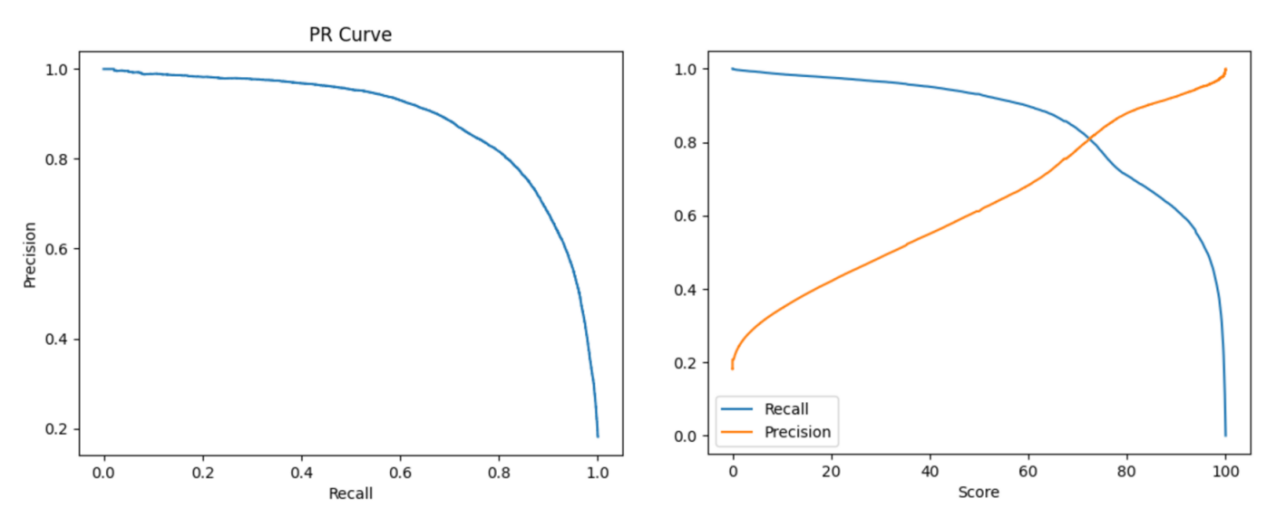


**Figure S1**. Precision and Recall curve of the De novo result


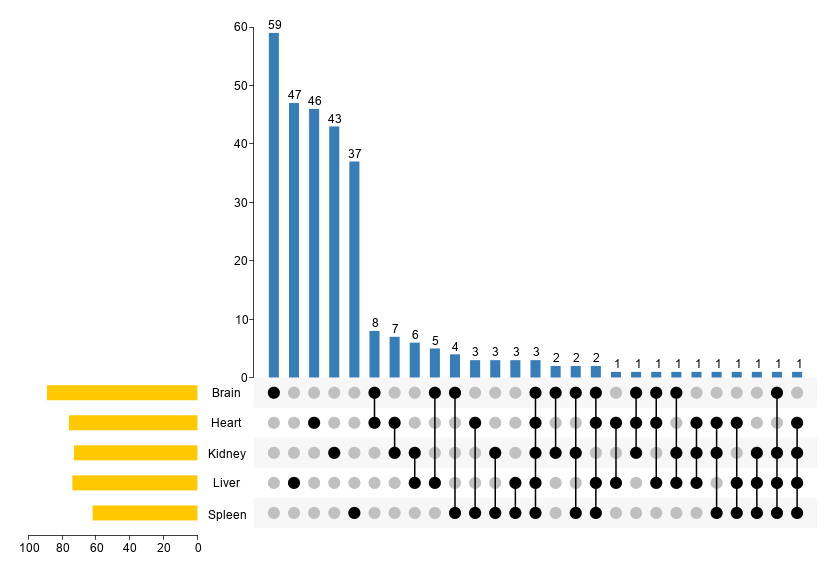


**Figure S2**. Distribution of detected microproteins in different tissues using de novo sequencing.


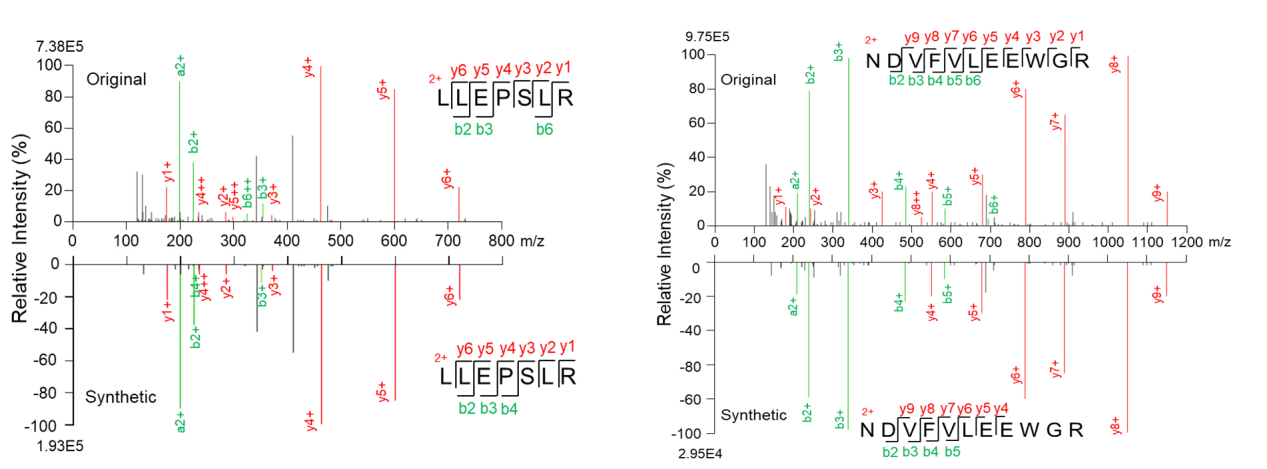


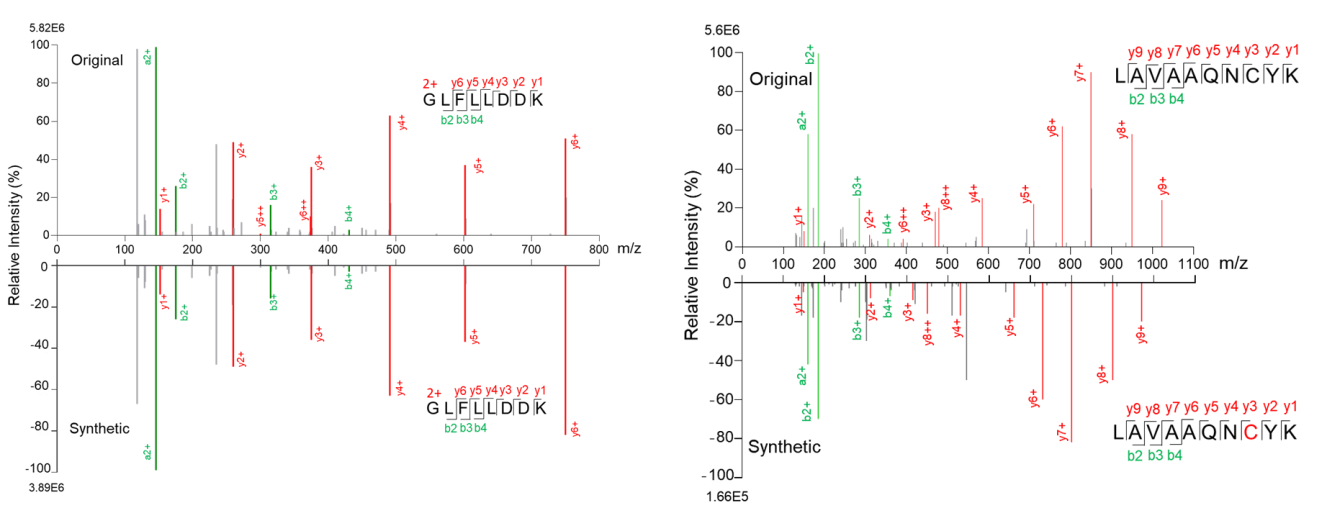


**Figure S3.** Comparison of the spectra for original de novo sequencing and synthetic peptides of LLEPSLR, NDVFVLEEWGR, GLFLLDDK, and LAVAAQNCYK.

**Figure S4.** The numbers of the microproteins and ncRNA-encoded microproteins among five tissues by top-down.

**Figure S5.** Comparison of the detected peptide sequence coverage among bottom-up, top-down, and de novo sequencing.


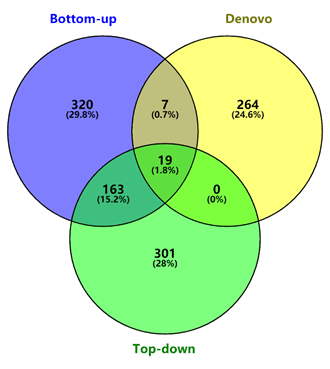


**Figure S6.** Venn diagram showing the number of the total microprotein identified using the three methods.


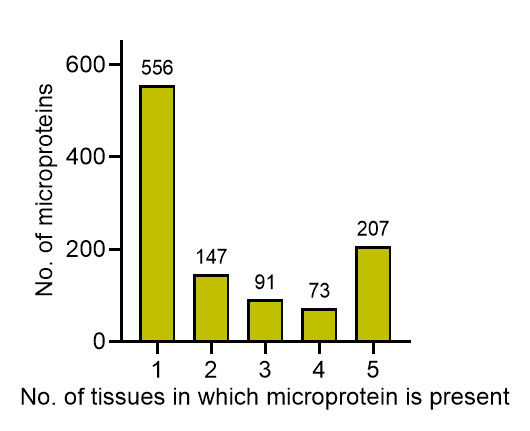


**Figure S7.** Tissue specificity of microproteins among five tissues.


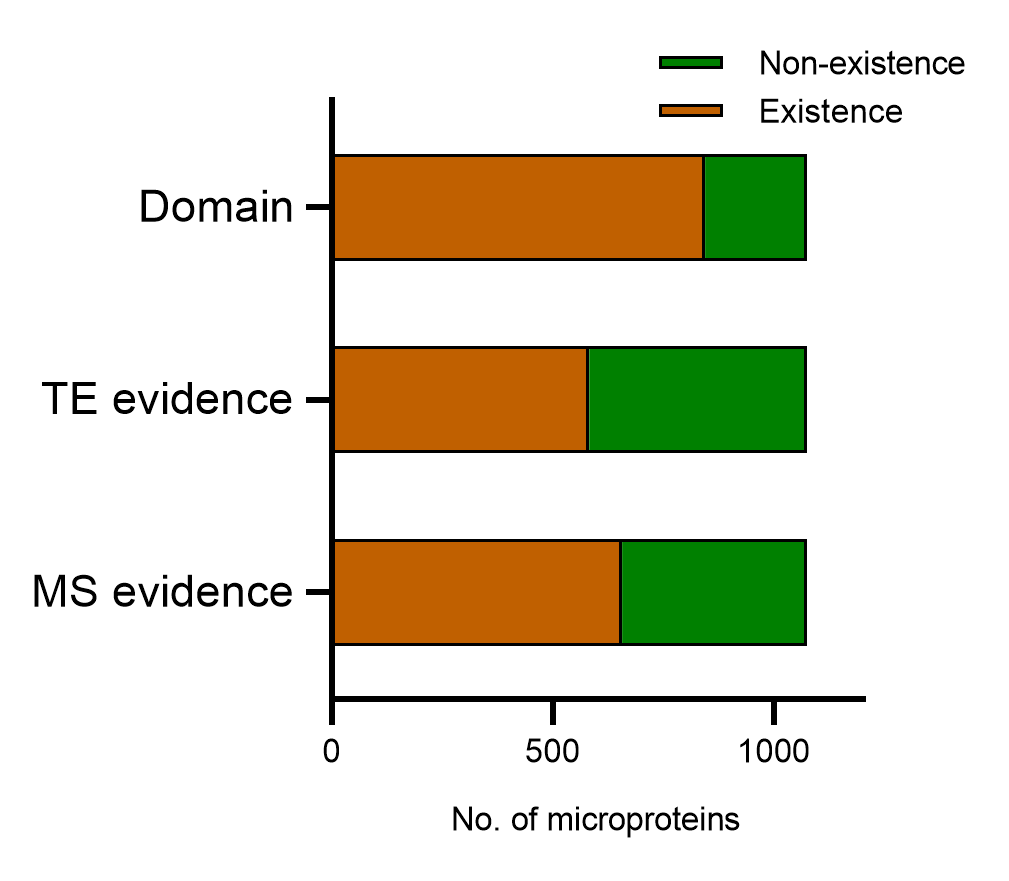


**Figure S8.** The proportion of microproteins with MS evidence, translation evidence, and domain.

**
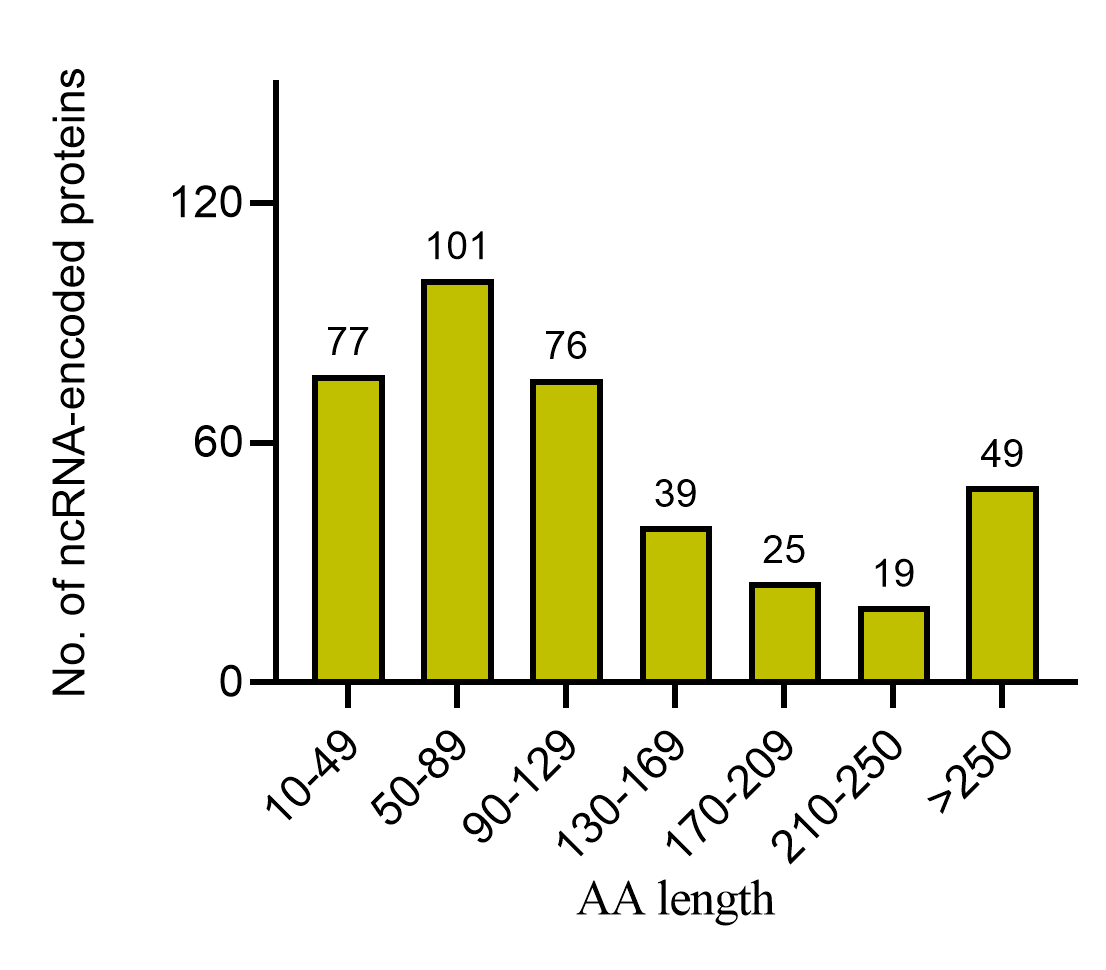
**

**Figure S9.** Length distribution of ncRNA-encoded proteins.


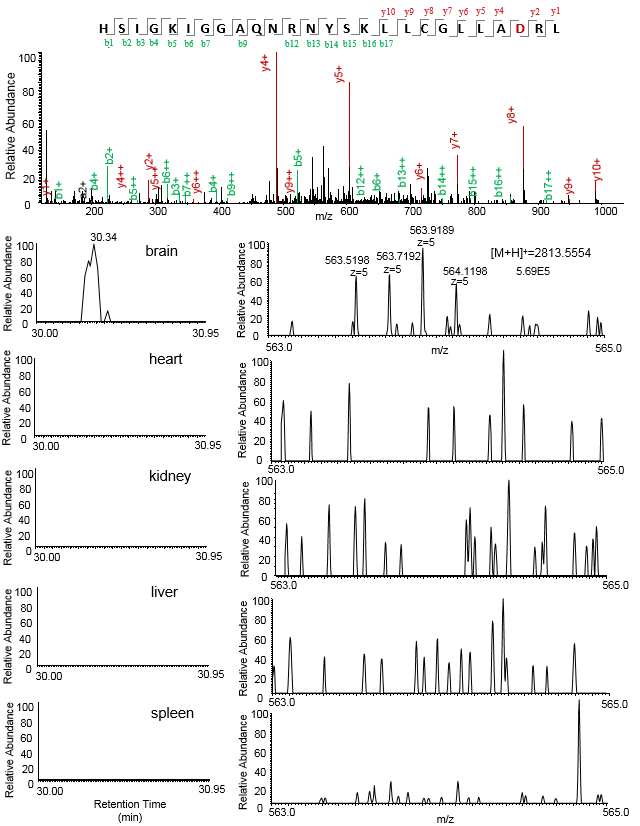


**Figure S10.** MS/MS spectrum (top), ion chromatographies (left) and precursor spectra (right) of microprotein IP_970184, which only identified in brain. Only brain sample can detect 3+ charged ion of this peptides, other samples didn’t detected this peptide at the same retention time.


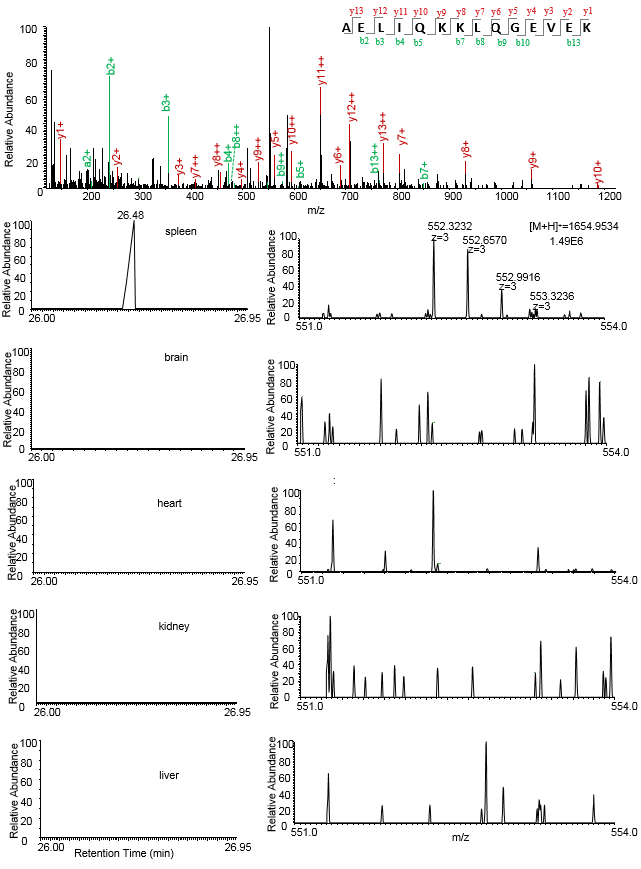


**Figure S11.** MS/MS spectrum (top), ion chromatographies (left) and precursor spectra (right) of microprotein IP_991787, which only identified in spleen. Only spleen sample can detect 3+ charged ion of this peptides, other samples didn’t detected this peptide at the same retention time.


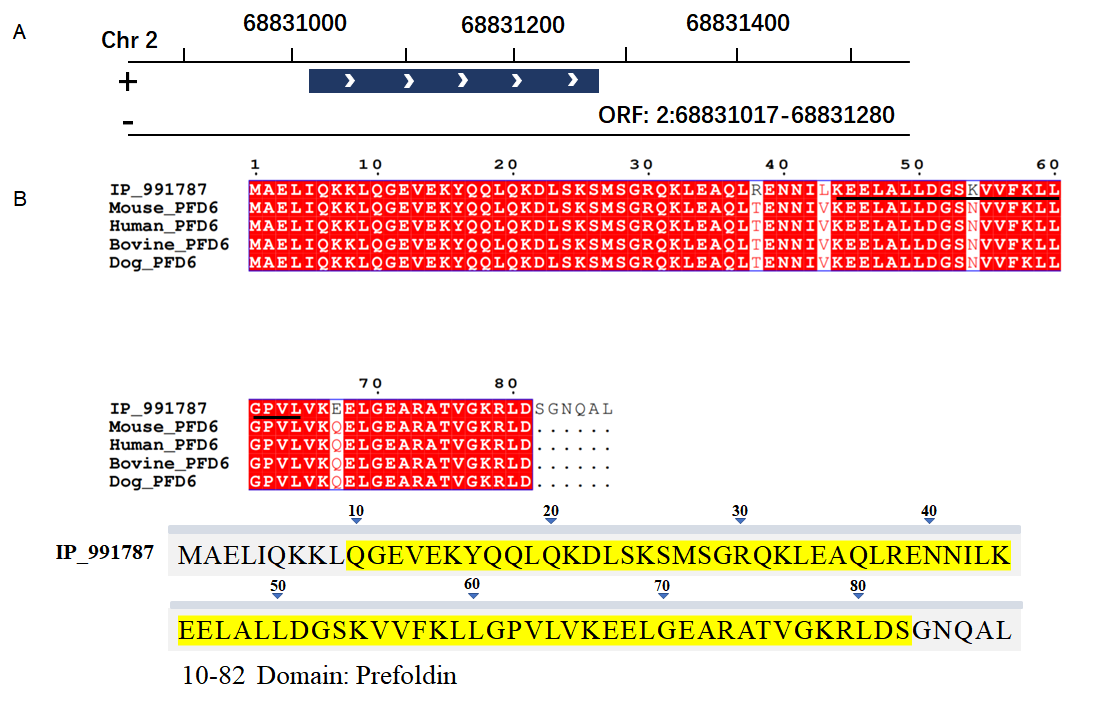


**Figure S12.** (A) Location of IP_991787 on mouse chromosome 2. (B) sequence alignment of IP_991787 and PFD6 across different species, the identified peptide is labeled with black line, and the domain sequence highlighted in yellow.
